# Supplementary material for: Molecular Correlates of Venous Thromboembolism (VTE) in Ovarian Cancer
Source: Cancers (Basel). 2022 Mar 15;14(6):1496. doi: 10.3390/cancers14061496 (PMC8946269; doi:10.3390/cancers14061496)
Supplement: Supplementary file 1 [file cancers-14-01496-s001.zip › cancers-1629711-supplementary/Figure S1 .pdf]

Article

Molecular Correlates of Venous Thromboembolism (VTE) in Ovarian Cancer

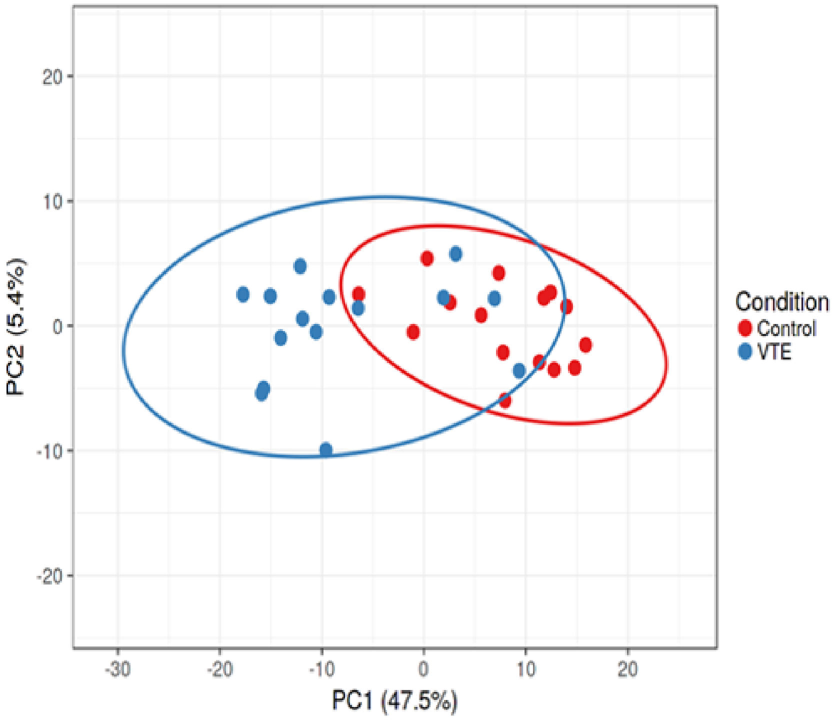

Figure S1: Principal component analysis of 255 significantly altered proteins in the VTE and control tumors.
